# Storage and Shelf-Life Assessment of Food Products

**DOI:** 10.3390/foods14162795

**Published:** 2025-08-12

**Authors:** Rui M. S. Cruz

**Affiliations:** 1 Department of Food Engineering, Institute of Engineering, Universidade do Algarve, Campus da Penha, 8005-139 Faro, Portugal; rcruz@ualg.pt; 2 MED-Mediterranean Institute for Agriculture, Environment and Development and CHANGE-Global Change and Sustainability Institute, Faculty of Sciences and Technology, Campus de Gambelas, Universidade do Algarve, 8005-139 Faro, Portugal

The assessment of food product storage and shelf life is a critical aspect of ensuring food safety, quality, and sustainability in the food industry. This entails a methodical assessment of numerous parameters that affect food-storage circumstances and the time frame over which the desired characteristics of a food product can be preserved. In today’s food industry, there is a growing focus on increasing shelf life while reducing the use of artificial additives and preservatives. Innovations in packaging technology, processing techniques, and natural preservatives have resulted from this focus [1,2,3]. The use of bioactive compounds and bio-based materials to produce effective and sustainable new packaging materials is a new trend to extend the shelf life of food products and help reduce the environmental impact of plastic materials [4,5,6]. Moreover, food processing and storage techniques aimed at the production of healthier, safer, and higher-quality food products and, consequently, their shelf life extension are another very important area in the food industry [7,8,9,10]. Therefore, the aim of this Special Issue was to present new challenges and new technological approaches related to the storage and shelf life of food products.

Zhao et al. (contribution 1) studied the effects of storage temperature (T1 (−18 °C) and T2 (−25 °C)) on the lipidomics profile in Pacific saury (*Cololabis saira*) for three months. The results showed that free fatty acids increased significantly; the content of triglyceride and phospholipid decreased; and the content of total cholesterol was maintained. Moreover, an increasing trend in the peroxide value, acid value, and content of thiobarbituric acid reactive substances was observed after the freezing process. The results showed that lipid oxidation was significantly higher in the group stored at −18 °C compared to the group stored at −25 °C. This study also identified 4854 lipid molecules in *C. saira*. Triglycerides (TG), phosphatidylcholine (PC), phosphatidylethanolamine (PE), phosphatidylinositol (PI), phosphatidylglycerol (PG), and diglycerides (DG) were among the lipid subclasses. The most prevalent lipid was TG, followed by PC. This work will contribute to the quality control of the frozen storage of pelagic fish.

Grigorakis et al. (contribution 2) assessed the quality of wild and farmed tropical fish species from national aquaculture and fisheries in the Saudi Arabian market and, subsequently, compared their freshness/deterioration. The studied fish species were either farmed, such as barramundi (*Lates calcarifer*), snubnose pompano (*Trachinotus blochii*), and sobaity bream (*Sparidentex hasta*), or caught in the wild, including cobia (*Rachycentron canadum*), coral trout (*Plectropomus leopardus*), giant trevally (*Caranx ignobilis*), milkfish (*Chanos chanos*), and mangrove red snapper (*Lutjanus argentimaculatus*). The results showed that fish shelf lives stored at 4 °C ranged from 8 (coral trout) to 18 days (sobaity bream). The wild-caught species always presented a significantly shorter shelf life than the farmed species. At the time of sensory rejection, the microbial load reached or exceeded a level of 6 log CFU/g while the K-values, depending on the species, ranged from 30 to 80%. The results obtained in this study contributed to freshness control and quality assurance during commercialization in local Saudi Arabian markets and the rest of the world.

The article by Ahmadi et al. (contribution 3) addressed the quality enhancement of stored chilled farmed rainbow trout (*Oncorhynchus mykiss*) using algal (*Cystoseira myrica* and *Cystoseira trinodis*) extract–ice combinations. The icing system was composed of ice containing different kinds of *C. myrica* and *C. trinodis* extracts during a 16-day chilled storage period. The results showed that all alga-treated fish presented lower pH values, as well as decreased lipid hydrolysis (measured through free fatty acid) and oxidation development (evaluated using the thiobarbituric acid index) compared to control samples. In terms of microbiological analyses (including aerobic, psychrophilic, Enterobacteriaceae, proteolytic, and lipolytic counts), most of the fish samples from the alga-treated batches presented lower values; preservation effect was particularly significant at longer storage times. Water and water–ethanol extracts showed greater inhibitory effects than their corresponding ethanol extracts. Furthermore, a higher total polyphenol content was identified in the water and water–ethanol extracts from both algae compared to those obtained solely with ethanol. This technique is an alternative to replace synthetic preservative compounds with those sourced from natural origins, aimed at producing high-quality seafood and food in general.

Custodio-Mendoza et al. (contribution 4) investigated targeted and untargeted carbonyl compounds and volatile organic compounds (VOCs) in adult formulas stored at three different temperatures (room temperature, 4 °C, and 60 °C) for one month. High-protein formulas presented the highest levels of carbonyl compounds, with 80% of samples showing levels of malondialdehyde, acrolein, and formaldehyde. 2-Heptanone and hexanal were also present in samples stored at higher temperatures. The results recommend that, after opening, storage should be performed in the original packaging, guaranteeing light, moisture, and heat protection. Formulas presenting high levels of protein should be kept refrigerated. These results contribute to the consumer safety and quality control of carbonyl compounds and VOCs in adult formulas.

Using gas chromatography–mass spectrometry (GC-MS) and a variety of bioassays, Verešová et al. (contribution 5) investigated the chemical composition and antimicrobial properties of *Rosa damascena* essential oil (RDEO) to determine its potential for food preservation. *Salmonella enterica* inoculated eggplant was treated with *Rosa damascena* essential oil. According to the GC-MS analysis, phenylethyl alcohol, an antimicrobial with aromatic properties, makes up 70% of RDEO. The minimal inhibitory concentration (MIC) was tested against five *Candida* yeast strains, four Gram-positive, and four Gram-negative bacteria, including biofilm-forming *Salmonella enterica*. MIC showed the strongest effects on Gram-negative species, with MIC50 values as low as 0.250 mg/mL for *S. enterica*. Moreover, an in situ assessment using kiwi and banana showed that the vapor phase of RDEO significantly suppressed microbial growth. RDEO was found to have insecticidal activity against *Megabruchidius dorsalis* (Fahraeus, 1839), an inhibitory effect on *S. enterica* during storage, and an antimicrobial effect on the microbiota of sous vide processed eggplant. Applications for RDEO, a natural antimicrobial agent, include food safety and preservation.

The article by Faria et al. (contribution 6) studied the use of locust bean gum/κ-carrageenan film with the extract of blueberry or beetroot as intelligent films for hake (*Merluccius merluccius*) freshness monitoring. At the end of storage, the BLE films’ color changed from pink to blue. According to the hake deterioration profile, these results are associated with pH values (from 6.60 ± 0.04 to 8.02 ± 0.03), total viable count (TVC, from 4.61 ± 0.36 to 8.61 ± 0.21 log CFU/g), and total volatile basic nitrogen content (TVB-N, from 10.21 ± 1.97 to 66.78 ± 4.81 mg/100 g) above the spoilage threshold. This study produced a novel bio-based responsive indicator film that incorporates a natural dye, BLE, which could help reduce food waste and enhance food safety.

Komeroski et al. (contribution 7) evaluated the post-harvest storage and shelf life of arugula microgreens (*Eruca sativa*) in different types of packaging (open air, vacuum sealed, and an under-modified atmosphere (MAP)) through microbiological, physico-chemical, and sensory parameters for 10 days at 5 °C. *Salmonella* spp., *Escherichia coli*, and *Listeria monocytogenes* were not observed in arugula microgreens, regardless of the day of storage and packaging used. On the other hand, the total Enterobacteriaceae was around 7 log CFU/g after 10 days of storage for all packaging systems. The results of the total count of mesophilic, psychrotrophic, and molds and yeasts were between 8 and 9 log CFU/g after 10 days of storage for all packaging systems. Open packaging proved to be promising, with less weight loss and slower chlorophyll degradation. The microgreens stored in the vacuum-sealed packaging showed a decrease in quality from the fifth day onwards for all attributes (sensory analysis). On the other hand, MAP showed good scores similar to the fresh microgreens (better visual quality).

In the article by Dermesonlouoglou et al. (contribution 8), the quality of osmo-dehydrofrozen cherry tomatoes was kinetically discussed during frozen storage. The optimum processing conditions were as follows: OD: T_OD_ = 36 °C, t_OD_ = 72 min, and C_glycerol_ + 61.5% *w*/*w*. This indicated water loss WL ≤ 5, color change ΔE ≤ 8, and reduced water activity (a_w_) at the end of the pretreatment. OD-pretreated frozen cherry tomatoes during frozen storage presented an acceptable color, an a_w_ decrease from 0.95 to 0.92, higher firmness, lower drip loss, and higher vitamin C/lycopene retention. Based on the loss of sensory quality, OD extended the shelf life of frozen cherry tomatoes by up to 3.5 times.

Chen et al. (contribution 9) studied the changes in the quality of kelp gel edible granules during storage. The results showed that the total bacterial counts were maintained within the national standard range, while the hardness and chewiness increased during storage at 4 °C. Conversely, during storage at 25 °C, the total bacterial count exceeded the national standard on the 20th day. Additionally, the gel strength, chewiness, and hardness all increased before declining. Significant water loss, decreased volume, a shift in color from bright green to dark yellow-brown, and a pronounced smell of decaying algae were observed in the product’s spoilage. Throughout the storage period, no coliform bacteria were found in any of the products. The product presented a 16-day shelf life at 25 °C, while this exceeded 20 days when stored at 4 °C. Storage at low temperature contributed to the product’s quality, maintaining its overall color.

The final article from de Medeiros et al. (contribution 10) is a review regarding the use of mucilage and bioactive compounds from *Cactaceae* to develop innovative and sustainable edible films and coatings that can preserve fruits and vegetables. Important production techniques, technological and functional characteristics, and the chemical composition were all updated in this review. Furthermore, the impact of these edible films and coatings on the fruits and vegetables, as well as market challenges and prospects, was presented.

Future research on new packaging technology, processing techniques, and natural preservatives will be needed to guarantee the quality and shelf life of food products.
